# T-type calcium channels trigger a hyperpolarization induced afterdepolarization in substantia nigra dopamine neurons

**DOI:** 10.1186/1471-2202-16-S1-P123

**Published:** 2015-12-18

**Authors:** Rebekah C Evans, Zayd M Khaliq

**Affiliations:** 1 NINDS, NIH, Bethesda, MD 20892, USA

## 

Dopamine neuron dendrites integrate synaptic information arriving from a diverse set of cell classes including excitatory and inhibitory neurons. The majority of synapses formed onto substantia nigra (SNc) dopamine neurons (>70 %) arrive from inhibitory GABAergic neurons [1]. Activation of GABA receptors pauses tonic firing and hyperpolarizes cells. Although the pause in dopamine neuron firing has behavioral relevance during reward omission [2], the hyperpolarization may also enable burst firing through disinhibition [3] or engagement of voltage-gated ion channels [4]. However, it is not clear how hyperpolarization influences dendritic integration. Here we use detailed single-cell computational modeling, patch-clamp electrophysiology, and two-photon calcium imaging to investigate the dendritic response to hyperpolarization. We find that at hyperpolarized potentials, some dopamine neurons respond to brief somatic current injections with a long-lasting depolarizing plateau that is accompanied by a large calcium transient in the dendrites. Because this depolarizing plateau requires prior hyperpolarization, we will refer to it as a hyperpolarization induced afterdepolarization (HI-ADP).

Using electrophysiology and calcium imaging, we found that pharmacological block of T-type calcium currents with TTA-P2 completely eliminated the dendritic calcium transient and greatly reduced the size of the HI-ADP. In contrast, blocking L-type calcium channels with nifedipine did not significantly alter the dendritic calcium or size of the HI-ADP. To further investigate the involvement of dendritic T-currents in generating the HI-ADP, we developed a multi-compartmental, multi-channel model of an SNc dopamine neuron in Genesis simulation software. This model contains a spherical soma with two dendritic trees extending from it, each containing identical primary, secondary and tertiary branches. Our simulations showed that a high density of dendritic T-type calcium channels is critical to the generation of the HI-ADP. Specifically, we found that reducing the T-type channel conductance by half throughout the entire cell completely abolished the HI-ADP. On the other hand, removing T-type channels from only one dendritic tree slightly reduced, but did not eliminate the HI-ADP. These simulations show that a high, localized density of T-type channels is more important to the generation of the HI-ADP than the total number of T-type channels in the cell.

Further simulations predict that the tightly coupled, electrotonically compact dendrites characteristic of SNc dopamine neurons are also necessary for the production of the HI-ADP. In particular, reducing the membrane input resistance of the model cell eliminated the HI-ADP. Confirming this prediction, our experimental data show that reducing the input resistance of SNc dopamine neurons through activation of G-protein coupled inwardly-rectifying potassium channels (GIRKs) abolished the HI-ADP and reduced the concomitant dendritic calcium transient.

In conclusion, we have shown that T-type calcium channels and electrotonically compact dendrites are essential for generating a HI-ADP in SNc dopamine neurons. These HI-ADPs represent an interesting response to hyperpolarization that may be unique to SNc dopamine neurons having the specific combination of high T-type channel density and tight electrotonically compact dendrites. This particular combination of characteristics may allow SNc dopamine neurons to respond to inhibition or the release of inhibition with an increased ability to burst.
